# Movement-Anchored Emotional Memory: An Embodied Neuropsychological Framework

**DOI:** 10.3390/jcm15186971

**Published:** 2026-09-09

**Authors:** Sharon Vaisvaser

**Affiliations:** School of Society and the Arts, Faculty of Humanities and Social Sciences, Ono Academic College, 1 Academic Avenue, Kiryat Ono 5545001, Israel; sharon.vaisvaser@ono.ac.il; Tel.: +972-50-7419912

**Keywords:** emotional memory, embodied cognition, sensorimotor processes, movement quality, memory reconsolidation, predictive processing, interoception, trauma, movement-based psychotherapy

## Abstract

This article advances a clinically oriented neuropsychological framework and introduces the construct of movement-anchored emotional memory, proposing that bodily movement constitutes a core organizing dimension of emotional memory encoding, reactivation, and transformation. Integrating embodied cognition, affective and social neuroscience, developmental psychology, trauma research, and neuropsychology, emotional memory is conceptualized as emerging through dynamic coordination among sensorimotor, interoceptive, affective, relational, and contextual processes. Movement contributes temporal, spatial, and action-related structure to emotional experience and memory, through bodily rhythms, movement qualities and sensorimotor sequencing that link immediate experience to prior states and anticipated action. In trauma, disruption of this temporal integration may reduce past–present differentiation, sustaining defensive patterns in current experience. The article reviews the embodied neural foundations of emotional memory and examines the developmental emergence of movement-anchored emotional memory, trauma-related memory alterations, and embodied pathways for memory updating. Within a regulated relational context, reactivation and variation of movement patterns may introduce new sensorimotor and affective information, generating mismatch between expectations and current experience, creating conditions for reconsolidation. The framework highlights movement as a pathway for accessing and reorganizing embodied aspects of emotional memory. Clinical implications for the embodied processing of emotional memory extend to trauma treatment, affect regulation, and movement-based therapeutic practice.

## 1. Introduction

Emotional memories emerge within ongoing patterns of affect, perception, physiological regulation, and action that unfold through continuous brain-body-environment interaction. Yet, while contemporary accounts increasingly recognize the embodied nature of emotional memory, the specific contribution of movement to its organization remains comparatively underdeveloped. Movement may constitute an important organizing dimension of emotional memory formation [1]. Emotional experiences unfold within ongoing patterns of bodily action and spatial configuration, which are intrinsically coupled with affective and physiological states. During encoding, these sensorimotor dynamics become integrated with exteroceptive perceptual and interoceptive signals, contributing to the formation of multimodal memory traces that coherently encompass bodily sensations, action tendencies, and affective meaning [2].

This framework draws on philosophical antecedents in phenomenology of embodiment, particularly Merleau-Ponty’s concept of motor intentionality [3]—the idea that the body is a field of intentional comportment, oriented toward the world through skilled, sedimented movement habits that pre-reflectively organize perception and action. Varela, Thompson, and Rosch [4] further developed this insight within the enactive cognition framework, arguing that cognition arises through embodied sensorimotor engagement with the world rather than through abstract manipulation of internal representations. Applied to emotional memory, these philosophical perspectives suggest that embodied movement habits and action tendencies may carry forward the residue of prior emotional experience not merely as stored representations but as living, enacted orientations toward the world—organizations that can be encountered, varied, and potentially transformed through new embodied experience.

Contemporary research in embodied cognition further indicates that cognitive and mnemonic processes are grounded in distributed sensory, motor, and affective systems through dynamic organism-environment interactions [5,6]. This 4E cognition perspective, encompassing embodied, embedded, enactive, and extended cognition, posits that cognition is shaped by the agent’s body, situated within its ecological niche, enacted through reciprocal sensorimotor engagement with the environment, and potentially distributed beyond bodily boundaries. These findings suggest that bodily states present during an experience participate directly in how memories are organized and later reconstructed via situated simulations [6,7].

Movement may therefore provide a scaffold through which emotional experiences unfold, become encoded within memory, consolidated and later recalled. Memory reconsolidation is a flexible process in which reactivated memories become temporarily labile and can be restabilized in updated form, allowing them to be strengthened, weakened, revised, or integrated with new information [8,9,10].

Because retrieval involves partial reinstatement of previously encoded bodily and sensorimotor states, movement may also provide a route through which emotional memories are reactivated and encounter new embodied information. This possibility is particularly relevant to contemporary accounts of memory updating and reconsolidation. This perspective shifts the understanding of memory from a predominantly representational model toward a dynamic, embodied process grounded in action and bodily engagement.

### 1.1. Aims and Rationale

Despite growing interest in embodied cognition, affective neuroscience, and memory reconsolidation, the specific contribution of bodily movement to the organization of emotional memory has remained comparatively underdeveloped. Contemporary accounts of emotional memory have advanced substantially in specifying the neural, interoceptive, predictive, and developmental dimensions of affective memory processes; yet these perspectives have rarely been synthesized within a shared framework that positions movement as a constitutive dimension of emotional memory encoding, reinstatement, and transformation. Likewise, developmental, trauma-related, and therapeutic perspectives on embodied memory have not been systematically integrated within a unified account that foregrounds the temporal, spatial, and action-related structure that movement contributes to emotional experience.

The present article addresses this gap by advancing the construct of movement-anchored emotional memory—an integrative neuropsychological framework in which bodily movement is proposed as a core organizing dimension of emotional memory formation, reactivation, and potential transformation. More specifically, movement-anchored emotional memory refers to the integration of dynamic movement-related features with affective, interoceptive, perceptual, contextual, and relational information during emotionally significant experience. Movement patterns, forms, and qualities accompanying lived experience, including posture, rhythm, spatial orientation and boundaries, muscle tone, temporal sequencing, and action readiness, are proposed to participate in encoding and later reinstatement of emotional memory, as well as in its potential updating within therapeutic and relational contexts. Within this formulation, movement includes overt bodily movement and subtle action-related bodily configurations; posture is included insofar as it carries affective and action-related organization, whereas motor imagery is treated as a related but distinct simulated sensorimotor process rather than as equivalent to enacted movement. Conceptually, emotional memory is the overarching category, referring to memory for emotionally significant experience; body memory denotes the broad persistence of prior experience in bodily dispositions, habits, and sensorimotor organization; and sensorimotor memory refers to learned perceptual-action regularities and motor patterns that need not be emotionally salient. Movement-anchored emotional memory is positioned more specifically at the intersection of these domains, emphasizing the integration of movement-related organization with affective, interoceptive, contextual, and relational dimensions of emotionally significant experience. This conceptual distinction is elaborated in Section 3.4. Integrating contemporary neuroscience with developmental, phenomenological, trauma, and psychotherapeutic perspectives, the framework examines how movement-anchored emotional memories may emerge through early sensorimotor-affective interactions, become disrupted or rigidified in the context of trauma, and be accessed and reorganized through embodied pathways of therapeutic change. In doing so, the article aims to advance a clinically oriented and multidimensional account of embodied emotional memory with implications for psychotherapy, trauma treatment, assessment, and movement-based clinical practice.

### 1.2. Integrative Scope and Literature Selection

This article presents an integrative narrative review aimed at synthesizing contemporary perspectives on movement-anchored emotional memory across embodied cognition, affective and social neuroscience, sensorimotor neuroscience, predictive processing, developmental psychology, trauma research, and movement-based clinical approaches. The literature discussed was selected through iterative, concept-driven searches across major scientific databases, including PubMed, PsycINFO, and Google Scholar. Search terms included combinations of concepts such as “emotional memory”, “embodied cognition”, “body memory”, “sensorimotor processes”, “dance/movement therapy”, “movement-based therapy”, “memory reconsolidation”, “predictive processing”, “interoception”, “peripersonal space”, “affect regulation”, “interpersonal synchrony”, “trauma”. Foundational phenomenological and psychodynamic texts were included based on their conceptual relevance to embodied and enactive accounts of memory and selfhood.

Searches covered literature available through August 2026; the final search was conducted on 29 August 2026. Records identified through these searches were screened by title and abstract for relevance to the framework, followed by full-text evaluation of potentially relevant sources; reference lists of key articles and reviews were also examined to identify additional foundational or recent studies. Inclusion was based on direct conceptual or empirical relevance to one or more core domains of the framework, while selection remained iterative and interpretive rather than exhaustive, consistent with the narrative review design.

Priority was given to peer-reviewed empirical studies, reviews, and theoretical contributions with direct conceptual or empirical relevance to the framework, with particular emphasis on literature addressing embodied emotional memory, sensorimotor processes, predictive processing, developmental origins, trauma-related alterations, and movement-based therapeutic mechanisms. Recent interdisciplinary research was integrated with foundational theoretical and developmental contributions to support the framework across its neuropsychological, developmental, and clinical levels. In areas where empirical findings are emerging or heterogeneous, particularly regarding movement-specific and clinical mechanisms, the manuscript adopts a heuristic and hypothesis-generating perspective and identifies directions for empirical investigation. Consistent with its integrative narrative design, the literature was synthesized qualitatively to identify converging findings, conceptual links, and testable propositions relevant to the proposed framework.

The review first examines the embodied neurobiological foundations of emotional memory and then considers how movement may contribute specifically to its encoding and reinstatement. It next traces the developmental emergence of movement-anchored emotional patterns within early relational experience and examines how these patterns may become rigid or insufficiently contextualized following trauma. Finally, it proposes a clinically oriented model in which movement-based reactivation, relational regulation, prediction error, and sensorimotor variation may contribute to emotional memory updating and reorganization.

## 2. Emotional Memory as an Embodied Brain-Body Process

### 2.1. Neural Substrates of Emotional Memory

Emotional memory emerges from distributed interactions among sensorimotor, limbic, paralimbic, and prefrontal systems. Central to these interactions is the functional coupling between the amygdala and hippocampus, both situated within the medial temporal lobe, through which the affective significance of experience modulates encoding, consolidation, and later retrieval [11,12]. This emotional enhancement of memory manifests as superior recall for affectively arousing events, supported by modulation of amygdala-hippocampal activity that selectively prioritizes emotional representations over neutral ones [13]. Neuromodulatory influences involving noradrenergic and glucocorticoid systems contribute to the enhanced encoding and stabilization of emotionally salient events, helping to account for the greater vividness and persistence of emotional compared with neutral memories [14,15]. At the same time, the hippocampus supports the contextual and spatial organization of these experiences, enabling emotional events to be embedded within coherent and broader episodic representations [16].

Emotional memory nevertheless extends beyond classical limbic memory circuitry. Regions including the anterior insula (AI), anterior cingulate cortex (ACC), medial prefrontal cortex (mPFC), and sensory and sensorimotor cortices contribute to the integration of interoceptive, perceptual, contextual, and action-related information during emotional experience [17,18]. Dynamic emotional states shape the episodic structure of memory, transforming ongoing experience into memorable events [19]. Evidence from neuroimaging and experimental studies indicates that somatosensory and motor cortices participate in representing emotional states, underscoring the embodied nature of affective experience [20].

Together, these findings position emotional memory as a distributed process in which affective significance, contextual information, bodily state, and action-related signals are integrated during lived experience. This distributed architecture provides the neurobiological basis for considering emotional memory as fundamentally embodied. These bodily dynamics may contribute to how emotional experiences are encoded and later retrieved [21,22].

### 2.2. Interoception and the Bodily Organization of Emotional Memory

Interoception provides a central pathway through which bodily states become integrated with emotional experience and memory. It refers to the sensing and integration of signals arising from within the body and contributes to emotional awareness, physiological regulation, and adaptive behavior [18,23,24]. Through interactions involving the AI, ACC, and connected cortical and subcortical systems (see Section 2.4), interoceptive information contributes to the subjective organization of affective states.

Importantly, bodily states are not merely consequences of emotional experience but participate in its encoding and later reconstruction. Emotional events are experienced within particular configurations of autonomic arousal, visceral sensation, proprioception, and action readiness, allowing bodily information to become integrated with perceptual and contextual features of the event. During later retrieval, partial reinstatement of these bodily states may contribute to the subjective reliving of emotional memories and facilitate access to related autobiographical content [25,26,27].

Recent work further suggests that bodily self-consciousness contributes to both the encoding and later reliving of autobiographical experience, with hippocampal and hippocampal-parietal mechanisms linking memory to embodied self-representation [28]. Emotional memory can therefore be understood as incorporating what has happened, how it was interpreted, and how the event was experienced from within a particular bodily state and bodily sense of self.

### 2.3. Predictive and Constructive Accounts of Emotional Memory

Predictive processing provides a complementary account of how bodily information becomes organized into emotional experience. The brain continuously interprets incoming bodily signals to generate predictions about internal states and guide adaptive responses to environmental demands [21]. Emotional experience can therefore be understood as dynamically constructed through interactions among bodily signals, contextual information, prior knowledge, and predictions about the person’s current and anticipated state [21,29].

Constructionist accounts similarly emphasize that physiological or affective changes do not in themselves constitute differentiated emotional experiences. Bodily states acquire emotional meaning through conceptualization and contextual interpretation [30]. Applied to memory, this suggests that emotional memories are not fixed reproductions of prior events but are reconstructed through the interaction of affective state, conceptual knowledge, bodily information, and contextual processing [31]. Emotional autobiographical memories likewise show a dynamic balance between stability and malleability, preserving meaningful aspects of past experience while undergoing reconstruction and transformation across repeated remembering [32].

This perspective is especially relevant to psychotherapy because reactivation of emotional memory necessarily occurs within a new present-moment context. The meaning attributed to reactivated bodily and affective states can therefore change as new contextual, relational, and experiential information becomes available [33].

### 2.4. Large-Scale Networks Linking Body, Memory, and Self

Emotional processing is interlocked with perception, cognition, motivation, and action, and these interactions are supported by the brain’s large-scale network architecture [11]. At the network level, emotional memory depends on coordinated interactions among large-scale systems that integrate bodily salience, autobiographical context, and sensorimotor action-related information [34,35,36]. The salience network (SN) plays a key role in detecting biologically relevant stimuli and coordinating autonomic and attentional responses. Interoceptive pathways, integrating visceral, proprioceptive, and vestibular signals, are embedded within the SN, anchored in the AI and ACC, and link bodily states with emotional appraisal and self-related processing [37]. Through its connections with limbic and brainstem systems, this network integrates internal bodily states with environmental cues, facilitating the detection of emotional significance.

At the same time, the default mode network (DMN) contributes to autobiographical memory, self-related processing, and the integration of emotionally significant experience across time [38]. Regions within the mPFC, posterior cingulate cortex, and hippocampal formation contribute to the contextual and autobiographical organization of emotionally significant experiences. Corticocerebellar networks complement these processes by integrating movement-related aspects of experience with affective and mnemonic processing through interactions with distributed cortical and subcortical systems [39]. Recent evidence further indicates that the cerebellum participates in social and emotional learning through reciprocal connections with prefrontal, limbic, and temporal regions, supporting prediction-error-based updating of social-affective sequences [40]. Cerebellar mechanisms have also been implicated in the temporal fidelity of emotional memory processes, including the consolidation and reconsolidation of fear-related memory traces [41]. The broader temporal dimension of movement-anchored emotional memory is further elaborated in Section 3.5.

The networks described above show stage-dependent shifts in relative engagement across emotional memory processing. During encoding, SN regions contribute to prioritizing emotionally relevant bodily and environmental information, while the cerebellum participates in emotional-memory enhancement and interacts with amygdala–hippocampal and cingulate regions [39]. During consolidation, hippocampal-cortical coupling, including DMN-related medial prefrontal and parietal regions, supports integration of newly encoded information. During retrieval, autobiographical remembering recruits DMN regions along with salience, and sensorimotor regions [42]. Emotional memory therefore emerges through dynamic coordination of neural circuits: salience-related processes identify what is relevant, sensorimotor and interoceptive systems instantiate how the event is bodily experienced and acted upon, and autobiographical networks situate that experience within the broader continuity of the self. This distributed organization provides the basis for asking more specifically how movement becomes incorporated into emotional memory—a question addressed in the following section.

## 3. Movement as a Constitutive Dimension of Emotional Memory

If emotional memory is constituted through coordinated affective, interoceptive, perceptual, and action-related processes, movement represents a particularly important yet comparatively underexamined dimension of this organization. Movement-related features are incorporated into the multimodal organization of an experience such that they form part of the encoded and potentially reinstated memory pattern. Indeed, building on the embodied and neurobiological foundations outlined above, movement can be conceptualized as a constitutive dimension of emotional memory organization, participating in the formation, retrieval, and transformation of emotional memory across time.

### 3.1. Movement as an Organizing Dimension of Encoding

Emotional experiences unfold through ongoing changes in posture, movement, spatial orientation, and action preparation that shape how emotionally significant events are experienced and encoded [43,44]. Emotions are intrinsically linked to action tendencies that prepare the organism for adaptive responses such as approach, withdrawal, protection, or affiliation, in response to changing environmental demands [45,46]. These action-oriented dynamics are related to approach-avoidance behavior that emerge during emotionally salient situations [47,48,49]. Experimental evidence further demonstrates that vertical movement dimensions, specifically upward versus downward bodily orientation, systematically modulate affective memory bias and autobiographical memory specificity in depressed individuals, indicating that movement configurations may function as contextual cues for mood-congruent retrieval [50].

Because emotional events are experienced within these bodily configurations, the motor patterns accompanying them may become integrated into the encoding of emotional memory [1]. Within the proposed framework, movement is hypothesized not merely to accompany emotional experience but to contribute to its encoding. by organizing temporal, spatial, and affective dimensions into integrated embodied patterns. Ongoing perception–action dynamics participate in the formation of the experience itself and, consequently, in the multimodal organization of its memory.

### 3.2. Spatial-Action Organization and Peripersonal Space

Emotional experiences unfold within a continuously organized action space surrounding the body, termed the peripersonal space (PPS). This PPS emerges through the integration of multisensory, motor, and affective information relevant for adaptive interaction with the environment [51,52]. PPS boundaries and organization are modulated by affective significance, interpersonal proximity, and possibilities for action. Emotional experiences therefore unfold not only within particular bodily states but also within particular spatial-action configurations, e.g., moving toward or away, expanding or contracting. Repeated associations among affect, bodily orientation, proximity, and action readiness may become incorporated into the organization of emotional experience and its later reinstatement, shaping self-experience across time [53]. By linking bodily states with action possibilities, PPS contributes to the spatial organization of emotional experience, shaping how affective events are experienced, encoded, and behaviorally enacted. As emotional experiences become associated with particular patterns of bodily orientation, proximity, and action readiness, these spatial-action dynamics may become incorporated into emotional memory traces [53].

Within predictive processing and active inference frameworks, affordances can be understood as dynamically organized possibilities for action, shaped by bodily capacities, motivational states, environmental constraints, prior experience, and anticipated outcomes. These action possibilities are integrated with hierarchical predictive processes and memory-based models of perception and action, allowing behavior to remain sensitive to both accumulated experience and current contextual demands [54,55].

As a theoretical extension of this evidence, emotionally significant experiences may contribute to the formation of predictive sensorimotor organizations linking bodily states, contextual cues, and anticipated action possibilities. When these memory organizations are later reactivated, they may reinstate affective and bodily states together with learned expectations regarding what actions are possible, necessary, or safe in the present context.

From this perspective, peripersonal space can be understood as a dynamically organized field of possible action shaped by bodily state, prior experience, and anticipated consequences of action. Its organization may therefore become part of the memory structure itself, such that reactivation of emotional memory also reinstates previously learned spatial and action-related expectations.

### 3.3. Sensorimotor Reinstatement During Memory Retrieval

Embodied and grounded cognition frameworks propose that memory retrieval entails partial reinstatement of the perceptual, motor, and affective states active during encoding [6]. From this perspective, remembering an emotional event may involve reactivation of aspects of the bodily configuration through which that event was originally experienced. Posture, spatial orientation and peripersonal boundaries, proprioceptive state, action readiness, and other sensorimotor features may therefore function as components of the retrieval context and contribute to the reconstruction of emotional experience.

Behavioral findings are consistent with this account. Bodily states can serve as contextual cues that facilitate access to congruent memories, and re-enactment or reinstatement of sensorimotor conditions associated with prior experience may influence what is retrieved and how it is subjectively experienced [56,57]. Thus, movement-related configurations may function as retrieval cues, enabling partial reinstatement of distributed sensorimotor-affective patterns associated with earlier experience.

Neuroimaging findings provide convergent, although indirect, support for this proposal. Somatosensory and motor cortices contribute to the representation of bodily and action-related aspects of emotional experience, and sensorimotor regions can be re-engaged during the retrieval or reconstruction of affectively significant information [58,59,60]. Such findings are compatible with the possibility that emotional memory retrieval involves reinstatement of bodily and action-related components of prior experience. However, direct evidence demonstrating the reinstatement of specific movement configurations during emotional memory retrieval remains limited. Direct neuroimaging evidence has shown modulation of sensorimotor circuits during the retrieval of negative autobiographical memories, providing further support for sensorimotor involvement in emotional memory retrieval [61].

### 3.4. From Body Memory to Movement-Anchored Emotional Memory

As outlined in Section 1.1, the proposed construct of movement-anchored emotional memory overlaps with, but is not identical to, the broader concept of body memory. Body memory has been used across phenomenological, cognitive, and neurorehabilitative traditions to describe ways in which prior experience continues to influence bodily perception, habits, dispositions, and sensorimotor organization without necessarily requiring explicit recollection [62,63]. This broad construct encompasses multiple forms of bodily continuity across experience and memory.

Movement-anchored emotional memory is proposed here as a more specific formulation. It refers to the integration of dynamic movement-related features such as posture, rhythm, movement quality, spatial orientation, sequencing, and action readiness with affective, interoceptive, perceptual, contextual, and relational information during emotionally significant experience. The emphasis is therefore not simply on the persistence of bodily experience, but on the dynamic organization of emotional experience through action across time and space. Indirect empirical support for the relevance of movement quality is provided by studies demonstrating that kinesthetic body feedback from differing movement qualities, such as light versus strong movement, produces measurable differential effects on affect and cognition, indicating that movement quality may function as a stored and reactivable component of emotional experience [64].

This distinction is clinically relevant. If movement-related organization constitutes part of an emotional memory pattern, then alterations in posture, rhythm, direction, spatial engagement, or action possibility may provide access to dimensions of experience that are not readily available through verbal or reflective recall alone. At this stage, however, this proposition should be understood as an integrative hypothesis derived from converging embodied-memory and sensorimotor research rather than as an established movement-specific memory mechanism.

The organization of movement-anchored emotional memory is also likely to vary across neurodevelopmental profiles. Autism provides a particularly relevant example, given evidence of substantial heterogeneity in sensorimotor processing, motor imagery and learning, interoception, and the neural organization of sensory experience [65,66]. These differences may shape how bodily and movement-related information is integrated with affective and contextual experience during encoding and later reinstatement. Importantly, similar emotional experiences may become organized through different sensorimotor configurations across individuals and neurotypes.

Taken together, converging behavioral and neuroimaging evidence suggests that emotional memories are encoded as multimodal patterns that include traces of bodily engagement with the environment. Within this broader embodied organization, movement may contribute a more specific temporal, spatial, and action-related structure through which emotional experience is encoded and later reinstated. These movement-anchored patterns emerge early in development and form foundational templates for later emotional experience.

### 3.5. Temporal Organization of Movement-Anchored Emotional Memory

Movement is organized not only through space but also through time. Bodily rhythms, including cardiac and respiratory cycles, proprioceptive flow, and sensorimotor sequencing, ground the sense of duration, continuity, and anticipation within ongoing action, with the bilateral insula, cerebellum, and supplementary motor area serving as convergence points for interoceptive and sensorimotor signals that represent temporal duration [67,68]. Phenomenologically, embodied experience carries a “temporally thick” structure, i.e., movement patterns integrate traces of the immediate past, engagement in the ongoing present, and anticipatory orientation toward the near future within a temporally extended present in which retention, present experience, and protention are continuously intertwined [53,69]. Because emotional experiences are encoded within these temporally organized movement patterns, movement-anchored memory traces carry not only spatial and affective dimensions but also implicit temporal organization.

Behavioral research demonstrates that preventing motor mimicry reduces or prevents the duration overestimation characteristic of emotional events; imagined motor activity may also modulate perceived duration, suggesting that movement patterns may shape the temporal frame within which emotional experience unfolds [70,71]. At the neural and phenomenological level, insular and interoceptive networks have been identified as the substrate through which bodily states constitute subjective duration, situating temporality within the ongoing stream of bodily life [72]. These findings provide direct evidence that motor engagement or simulation can alter temporal perception, while their relevance to movement-anchored emotional memory remains indirect and mechanistic.

The temporal organization carried within movement is particularly relevant to emotional memory, where the temporal situating of an experience, its belonging to a particular past context rather than to the ongoing present, depends in part on this embodied temporal structure. When emotional memory remains well-integrated, movement-anchored emotional configurations retain their temporal situatedness: reactivation occurs within a framework that contextualizes the experience as past while preserving access to its affective and sensorimotor dimensions. Disturbances in embodied temporal integration may therefore contribute to difficulties in maintaining this past–present differentiation, with implications for how traumatic experience is organized and re-experienced in the body.

## 4. Developmental Origins of Movement-Anchored Emotional Memory

### 4.1. Early Sensorimotor-Affective Coupling

From early development, affective experiences are organized through patterns of bodily movement, posture, and rhythm, within interaction with caregivers and the surrounding environment. These sensorimotor-affective exchanges provide a primary medium through which arousal, emotional significance, and relational experience are coordinated [73,74,75]. Repeated couplings between bodily states, movement dynamics, and interpersonal responses may therefore contribute to the formation of early embodied expectations linking affect, action, and relational meaning [76]. These developmental findings provide evidence for early sensorimotor-affective coupling; interpreting such coupling as a developmental basis for movement-anchored emotional memory represents a theoretical extension of the present framework.

These embodied patterns may later serve as bridges to implicit emotional memories, allowing affective experiences to be accessed and reactivated through movement-related cues and bodily states [62,77]. In this sense, movement may function as a privileged pathway to early movement-anchored emotional memories that were formed prior to language and are retained primarily in sensorimotor, affective, relational forms [76,78].

### 4.2. Co-Regulation, Synchrony, and Implicit Relational Expectations

Early sensorimotor-affective experience is shaped within repeated cycles of interpersonal contingency, synchrony, rupture and repair. Caregivers dynamically adjust their behavior to infants’ changing attentional, affective, and bodily states, creating temporally organized exchanges through which regulation and communicative expectations gradually emerge [79]. Recent evidence further conceptualizes parent-infant synchrony as a dynamic and multimodal process involving coordination across behavioral, physiological, and neural levels [80].

Direct neuroimaging evidence for this neural dimension of synchrony comes from hyperscanning studies in caregiver-infant dyads during naturalistic interaction, consistently documenting inter-brain coupling during conditions of mutual engagement [81,82]. This coupling is amplified by touch, close proximity, and temporally structured turn-taking [83], and covaries with caregiving quality [82]. Longitudinal data suggest that sensitive early care tunes a frontotemporal interbrain network whose organization remains detectable in adolescence [84], implying that early neural synchrony may represent an initial neurobiological substrate for the consolidation of relational and affective patterns into movement-anchored emotional memory.

Across these recurrent interactions, infants gradually develop expectations regarding how arousal can be regulated, how others are likely to respond, and what forms of proximity, withdrawal, approach, or engagement are available within the relationship. Repeated movement, touch, rhythm, and spatial coordination may therefore contribute to implicit relational organizations linking bodily states with affective meaning and anticipated interpersonal outcomes. Dance/Movement Therapy (DMT) approaches engaging these relational and embodied dimensions have been applied to intergenerational transmission of trauma, illustrating how movement-based relational work may support the reorganization of embodied relational expectations carried across generations [85].

From a predictive perspective, these embodied exchanges may contribute to developing expectations about bodily safety, interpersonal availability, and possible action. Over time, recurrent patterns of co-regulation may become increasingly stable sensorimotor-affective organizations that shape how subsequent relational situations are perceived and enacted. Repeated caregiving experiences may become consolidated into interpersonal affective or attachment schemas that support predictive inferences about emotion regulation and later social experience, with the mPFC proposed as a key substrate for the abstraction and use of such relational knowledge in mentalizing [86,87]. In this sense, early relational experience provides a developmental context in which movement, affect, and interpersonal prediction become progressively intertwined.

### 4.3. From Early Embodied Patterns to Movement-Anchored Emotional Memory

Early embodied organizations have been described clinically as enduring motor-affective patterns through which relational experience is carried forward across time. Shahar-Levy conceptualized such configurations as “emotive memory clusters,” referring to psychophysical organizations in which movement, affective states, and relational experience are tightly interwoven [78]. These clusters can be understood as early examples of movement-anchored emotional memory, recurrent configurations of posture, muscle tone, rhythm, spatial orientation, and action readiness that become associated with particular affective and relational meanings.

Because many of these patterns are formed before mature verbal and autobiographical memory systems are established, they may later be expressed primarily through bodily sensations, action tendencies, movement qualities, and relational responses rather than through explicit narrative recall. Their re-emergence in present-moment interaction may therefore reflect partial reinstatement of earlier sensorimotor-affective organizations shaped through repeated experience.

This clinical formulation converges with embodied accounts in which early experience is organized through perception–action processes and remains capable of influencing later bodily dispositions, habits, and relational orientations [77,88,89]. Such continuity implies recurrent sensorimotor-affective interactions may shape enduring patterns of expectation and responding that are re-enacted within subsequent bodily and relational contexts.

When early relational environments are characterized by repeated threat, neglect, or inconsistent regulation, the sensorimotor-affective organizations developing within these interactions may become increasingly organized around protection, vigilance, withdrawal, or constrained engagement. Childhood maltreatment has been associated with alterations in neural systems involved in emotion processing, sensory integration, attachment, and stress regulation, with effects that vary according to developmental timing and type of adversity [90,91]. Recent evidence also suggests that childhood maltreatment may affect aspects of bodily self-processing, including reduced trust in bodily signals, particularly following emotional maltreatment [92]. Within the present framework, repeated developmental adversity may therefore shape movement-anchored emotional organizations in which bodily states, relational expectations, and action possibilities become calibrated to environments characterized by unpredictability or threat.

In the context of overwhelming or traumatic experiences, these embodied memory traces may remain insufficiently integrated into higher-order cognitive systems, persisting as fragmented sensorimotor patterns that are re-experienced as present-moment bodily states rather than as past events [93]. Consistent with this developmental account, childhood trauma has also been associated with alterations in autobiographical memory, including reduced specificity and differences in the organization and accessibility of personal memories [94].

Together, these perspectives suggest that early emotional memory may be understood as fundamentally movement-anchored, emerging as dynamic configurations of embodied interaction that organize affective experience, relational expectations, and the developing sense of self. While the preceding sections outlined how emotional memory is formed within embodied systems, the following section examines how differences in the organization and integration of these components shape distinct memory expressions, particularly in the context of trauma.

## 5. Alterations in Movement-Anchored Emotional Memory in Trauma

### 5.1. Trauma as Disrupted Contextualization and Predictive Rigidity

Emotional memory varies in the degree to which affective, bodily, sensorimotor, contextual, and autobiographical components remain flexibly integrated. In autobiographical memory, bodily sensations, action tendencies, and perceptual features are integrated within temporally and contextually organized representations, allowing past experiences to be reactivated while remaining situated as belonging to the past [95]. Distortions in autobiographical memory recollection, however, appear across diagnostic categories and have been identified as a transdiagnostic feature of mood, anxiety, stressor-related, eating, and psychotic disorders [96]. These distortions include heightened accessibility and affective impact of negatively valenced memories, intrusive recollections, and reduced specificity of voluntary autobiographical retrieval. Trauma-related remembering involves a weakening of contextual and temporal integration, contributing to bodily and action-related responses that retain a strong sense of present relevance.

At the neural level, trauma-related memory disturbances have been associated with altered interactions among the amygdala, hippocampus, ACC, and prefrontal regulatory systems. Post-traumatic stress disorder (PTSD) has frequently been associated with heightened amygdala responsivity to threat-related cues, alterations in hippocampal processes supporting contextual discrimination, and disrupted recruitment and functional coordination of medial and ventromedial prefrontal regions involved in threat regulation and extinction [97,98,99]. Such changes may contribute to the persistence and generalization of threat responses, weakened differentiation between past and present contexts, and reduced updating of previously learned fear associations.

Accordingly, in PTSD, the crosstalk within and between networks has been shown to be disrupted: the SN exhibits increased within-network connectivity, while within-DMN connectivity is diminished and between-network SN–DMN connectivity is altered, reflecting a breakdown in the complementary relationship between salience detection and autobiographical contextualization [100]. During traumatic memory retrieval, DMN-sensorimotor network hyperconnectivity occurs alongside reduced functional connectivity in the cerebello-thalamo-cortical axis, which ordinarily contributes to temporally precise predictive coordination [101]. In addition, stress-induced increases in SN-cerebellar coupling have been associated with greater intrusion symptoms [102]. These findings provide a neurobiological rationale for considering bottom-up, sensorimotor-based approaches in traumatic memory reprocessing [101].

From a predictive processing perspective, trauma-related memory may involve persistent, highly weighted expectations of threat that continue to shape perception, bodily states, and behavior despite changes in the current environment [103,104]. Such threat-related predictions may bias the interpretation of incoming sensory information and sustain defensive patterns of vigilance, withdrawal, freezing, and action readiness [105].

From a movement-anchored perspective, these persistent threat expectations may also be expressed through recurrent patterns of bodily preparation and action. Posture, spatial orientation and peripersonal boundaries, muscle tone, and readiness to approach, withdraw, freeze, or protect may be reactivated in response to cues associated with earlier threat, even when the present context allows different forms of action. In this way, traumatic memory may continue to shape how the body is organized for action in the present. Evidence from trauma research directly supports alterations in contextualization, threat processing, and action readiness; the proposed reactivation of specific movement configurations constitutes a framework-based interpretation that remains to be tested directly.

### 5.2. Embodied Re-Experiencing and Temporal Context

Within the context of trauma, the temporal organization of movement-anchored memory described above undergoes disruptions. Movement patterns organized around past threat may fail to retain their temporal situatedness, contributing to characteristic re-experiencing phenomena in which past events are enacted as though presently occurring.

Trauma-related remembering may involve reduced differentiation between bodily responses associated with the past event and those relevant to the present context. Sensory, affective, and action-related components can be reactivated with considerable immediacy, contributing to bodily sensations, perceptual fragments, and action tendencies that are experienced as currently relevant [93,106]. This diminished contextualization is particularly relevant to movement because action systems are inherently oriented toward immediate and anticipated interaction with the environment.

From a movement-anchored perspective, reactivation of defensive motor readiness may therefore contribute to the experiential immediacy of traumatic memory. A posture of protection, a tendency to withdraw, muscular tension or armoring, or restricted spatial engagement may function as part of the present enactment of previously learned threat organization. The remembered experience may therefore be expressed through ongoing patterns of bodily preparation and action, alongside fragmented sensory, affective, and imagistic elements that remain insufficiently integrated into autobiographical narrative. Clinical observations in Holocaust survivors similarly suggest that traumatic memory may be carried and expressed through enduring movement and postural patterns beyond verbal recall [107].

Within a predictive processing framework, this temporal disruption can be understood in terms of overweighted threat predictions that narrow the range of perceived affordances and bias action selection toward repetitive defensive patterns [53]. When the peripersonal action field is organized around persistent threat, the forward-oriented protention that normally opens the present moment toward new possibility is instead captured by a loop of anticipated threat, what might be described as a form of embodied temporal freezing. Movement patterns originally organized around survival responses thus continue to function as present-tense predictions rather than past-tense traces, maintaining the experiential immediacy of the original event.

These processes may take different forms across trauma types. Following discrete traumatic events, re-experiencing may involve relatively circumscribed sensory, affective, and action-related fragments linked to a particular event. In developmental or relational trauma, repeated early experiences of threat, neglect, or dysregulation may contribute to broader and more enduring disruptions in autobiographical memory and in the integration of bodily and affective experience [90,91,92,94]. Relational disruption may extend to a more fundamental disturbance in the temporal continuity of embodied self-experience, whereby sensorimotor-affective experience remains insufficiently organized within a coherent sequence of meaningful events, thus undermining the sense of ownership and agency [53].

This developmental pattern resonates with Winnicott’s observation that early failures in continuity of being (“going-on-being”) lead experiences to recur as enacted states rather than remembered events [108], and with Bion’s characterization of unprocessed beta-elements as persisting outside symbolic thought and temporal sequencing [109]. From a movement-anchored perspective, these formulations describe enduring patterns of bodily preparation and action readiness that remain insufficiently integrated within autobiographical and temporal context, and are therefore re-enacted in the present.

### 5.3. Bodily Self, Agency, and Action Possibilities

Trauma-related alterations in bodily self-consciousness may further shape the relation between emotional memory and action. Recent predictive accounts emphasize disturbances in the integration of interoceptive and exteroceptive information in PTSD, with consequences for body ownership, bodily self-representation, and sense of agency [105]. In non-dissociative PTSD, hyperprecise trauma-related priors may contribute to rigid bodily self-representations and reduced perceptual updating, whereas dissociative presentations may involve a different weighting of interoceptive, exteroceptive, and prior information.

These alterations are particularly relevant to movement-anchored emotional memory because agency depends partly on the capacity to anticipate, initiate, and experience one’s actions as effective within the environment. When emotional experience has repeatedly been organized around threat, helplessness, or constrained action, later memory reactivation may be accompanied by a narrowed sense of what the body can do and which actions are likely to be effective. In predictive terms, this may correspond to a narrowing of perceived affordances, such that the range of actions experienced as available, safe, or effective becomes constrained by trauma-related expectations. Such patterns may influence both movement organization and the subjective sense of agency in the present.

Taken together, trauma-related emotional memory can be understood as involving altered coordination among contextualization, bodily state, predictive processing, and action organization. Within a movement-anchored framework, persistent sensorimotor-affective patterns may carry forward learned expectations concerning threat, proximity, bodily readiness, and available action. Therapeutic change may therefore depend on creating conditions in which these established expectations can be reactivated while new bodily, relational, and action-related possibilities become available.

## 6. Embodied Pathways and Mechanisms of Change in Working Through Emotional Memories

### 6.1. Accessing Movement Patterns in the Therapeutic Process

Embodied work with emotional memory involves processes in which patterns associated with prior experience become enacted in the present, brought into awareness, and gradually expanded or reorganized through embodied engagement within a safe relational context. Emotional memory may emerge through the sensorimotor-affective configurations described above, reflecting implicit modes of organizing experience. Such movement expression may occur across a wide range of amplitudes, from larger whole-body actions to subtle shifts, including changes in posture, weight distribution, breathing-related movement, hand and finger gestures, facial expression, gaze, and variations in muscular tension. These small-scale movement phenomena may carry affective and relational significance and provide clinically meaningful access to emerging patterns of embodied experience. Through guided or spontaneous movement, these patterns can become accessible to observation, feeling, and differentiation, while their enactment in the present generates sensorimotor and interoceptive input that renders implicit organization available for modulation. This activation does not necessarily involve the explicit retrieval of discrete memory content, but may instead involve embodied dynamics carrying affective and relational significance.

Recognition of these patterns within a regulated interpersonal field enables sustained engagement without overwhelming the individual. Conditions of safety and attunement support modulation of physiological arousal and the integration of bottom-up sensory signals with ongoing contextual processing [93].The clinician may engage with these patterns through attunement, mirroring, modulation, or the introduction of new movement possibilities. The therapist may also function as an attuned witness to emerging bodily and affective experience, supporting its recognition, differentiation, and integration within the therapeutic relationship. Within this relational context, bodily and affective experience can become increasingly tolerable, differentiated, and linked to memory and meaning.

These relational and somatic processes find clinical precedent in established embodied therapy approaches. One of the most prominent of these is DMT, which uses spontaneous and guided movement, mirroring, improvisation and internally focused movement exploration within a relational therapeutic frame to support the expression, differentiation, and integration of embodied emotional experience [110]. Sensorimotor Psychotherapy explicitly incorporates attention to movement, posture, gesture, and bodily sensation as primary therapeutic data and works with sensorimotor sequences to support the completion and integration of interrupted defensive responses [111]. Somatic Experiencing tracks bodily sensations and movement impulses associated with overwhelming experience, facilitating discharge and resolution of threat-related activation [112]. Although these approaches explicitly foreground bodily and movement-based processes, attention to bodily experience, action tendencies, affect regulation, and relational enactment may also be incorporated within psychotherapy across theoretical orientations. Across these approaches, the therapeutic relationship and co-regulatory attunement provide the relational container within which movement-based processes can be safely engaged and integrated.

### 6.2. Mismatch, Prediction Error, and Reconsolidation

Once identified and sufficiently regulated, movement patterns may be amplified, slowed down, varied, contrasted, or reorganized through changes in rhythm, force, sequencing, or spatial configuration. Repetition may support stabilization and recognition of the pattern, whereas variation introduces flexibility and facilitates the emergence of alternative modes of experiencing and responding. At the clinical level, movement variation is therefore proposed first as a candidate mechanism for emotional memory updating, through which new sensorimotor, affective, and relational information may be introduced during reactivation.

Prediction error and reconsolidation are considered possible mechanistic pathways through which such updating might occur. Importantly, the proposed link between movement-pattern reactivation, prediction error, and reconsolidation is a theoretical extension of established reconsolidation findings; direct experimental evidence for this movement-specific pathway is not yet available. From a predictive processing perspective, such variation may enable the updating of prior embodied expectations when current sensory and relational experience differs from what the activated pattern predicts. New movement possibilities may therefore introduce forms of embodied mismatch or prediction error, allowing previously established sensorimotor-affective expectations to encounter alternative bodily, relational, and action-related outcomes.

When novel embodied experiences occur while previously encoded emotional patterns are actively re-engaged, conditions may be created for memory updating and, under appropriate conditions, reconsolidation. Newly generated sensorimotor, affective, and relational information may then become integrated into the reactivated memory organization, potentially supporting more enduring transformation [113,114].

Importantly, there are specific conditions under which reactivation can lead to reconsolidation, rather than mere retrieval. Research indicates that reconsolidation requires a memory reactivation event that introduces a sufficient mismatch between what the reactivated memory predicts and what is currently experienced; without this prediction error, the memory may simply be retrieved without destabilization, leaving it unmodified [8,10]. The nature and extent of mismatch may influence the fate of a reactivated memory: insufficient prediction error may fail to destabilize the existing memory, whereas more extensive new learning may favor processes such as extinction rather than reconsolidation [115]. The time window for restabilization is also bounded, typically within several hours following reactivation. Within a movement-anchored framework, these boundary conditions suggest that therapeutic movement variation may need to be calibrated to generate embodied mismatch that is distinct but tolerable, introducing new sensorimotor possibilities that diverge meaningfully from established bodily predictions while remaining within the individual’s regulated window of tolerance [116,117]. Memory updating is used here as the broader term for modification of a reactivated memory representation. Reconsolidation refers more specifically to modification of the reactivated trace following destabilization and preceding restabilization, whereas extinction involves new inhibitory learning that competes with, rather than necessarily alters, the original memory. Evidence for these distinctions and boundary conditions derives primarily from basic experimental memory research; evidence for their operation in clinical populations is more limited, particularly for movement-based interventions in trauma treatment.

Reorganization may occur as newly generated patterns become sufficiently coherent and regulated to be integrated into broader self-related processing. This process may involve changes in how affect, bodily states, spatial orientation, and action tendencies are coordinated, supporting greater flexibility in responding to internal and external demands. Movement-based work may thereby expand the range of actions experienced as possible, allowing previously constrained patterns to become associated with alternative possibilities for approach, withdrawal when adaptive, boundary setting, exploration, or interpersonal engagement.

### 6.3. The Relational Field and Embodied Co-Regulation

These processes unfold within a relational field in which interpersonal attunement, co-regulation, and adaptive variation in movement and affective expression support the establishment and stabilization of new patterns. Such processes may involve interpersonal synchrony across behavioral, physiological, and neural levels. In psychotherapy, coordination of nonverbal behavior and bodily rhythms, together with emerging evidence of interbrain synchrony, has been associated with therapeutic alliance and moment-to-moment affective attunement [118,119]. Within a movement-anchored framework, such interpersonal coordination may provide a relational context in which reactivated sensorimotor-affective patterns can be experienced differently, allowing established bodily expectations to encounter new interpersonal and regulatory contingencies.

Through repeated cycles of activation, regulation, variation, and integration, movement-based therapeutic processes may support the gradual reorganization of emotional memory as it is enacted in the present.

Taken together, movement may function as a medium through which embodied aspects of emotional memory are accessed, expressed, differentiated, and reorganized within a safe and relationally attuned context. Such changes may contribute to greater affect regulation, flexibility, agency, and continuity of experience.

## 7. Summary and Conclusions

This article advances a clinically oriented neuropsychological framework of movement-anchored emotional memory, positioning bodily movement as a constitutive dimension in the encoding, reactivation, and potential transformation of emotional memory across time. Integrating embodied cognition, affective and social neuroscience, developmental psychology, trauma research, and contemporary neuropsychology, the framework conceptualizes emotional memory as emerging through dynamic coordination among sensorimotor, interoceptive, affective, relational, and contextual processes.

Three key contributions emerge from this framework. First, movement is conceptualized as an organizing dimension of emotional memory through which temporal, spatial, and action-related aspects of experience become integrated with affective and bodily states. Movement patterns accompanying lived experience may therefore contribute to how emotional experiences are encoded and later reinstated. The temporal dimension of movement extends to the organization of the perceived present moment that grounds emotional experience in time. Disruption of this temporal organization in trauma-related memory may reduce past-present differentiation, contributing to the persistent present relevance of prior bodily–emotional configurations. This perspective extends broader embodied accounts of memory by emphasizing the dynamic organization of experience through action across time and space.

Second, the framework traces the developmental origins of movement-anchored emotional memory to early sensorimotor-affective interactions. Recurrent sensorimotor-affective coordination with caregivers may contribute to embodied relational expectations linking bodily states, affective meaning, and anticipated possibilities for action.These early organizations may later be expressed through bodily sensations, action tendencies, movement qualities, and relational responses, particularly when explicit autobiographical access is limited.

Third, the framework considers movement variation within a regulated relational context as a potential pathway for emotional-memory updating. Prediction error and reconsolidation are proposed as possible mechanisms through which such updating may occur.

Trauma-related memory provides a particularly relevant context for this framework. Persistent threat-related predictions may continue to shape bodily states, perception, and action despite changes in the present environment, while reduced temporal and contextual differentiation may contribute to the continued present relevance of bodily and action-related aspects of past experience. Within a movement-anchored perspective, this sensorimotor-affective organization may constrain the range of actions experienced as available or effective in the present.

Clinical implications extend across neuroscience-informed psychotherapy, trauma treatment, assessment, and movement-based practice. These implications can be translated into four practical principles: (1) Observe movement patterns as they emerge within therapeutic interaction, to access embodied aspects of emotional memory that are only partially available through verbal reflection; (2) Characterize recurrent, person-specific sensorimotor-affective patterns to support their differentiation, and use this characterization to guide the selection and pacing of embodied interventions; (3) Within a responsive relational context, use the therapist’s bodily attunement, rhythm modulation and mirroring as a relational scaffold, alongside movement variation and new action possibilities calibrated to diverge meaningfully from established sensorimotor patterns while remaining within the individual’s window of tolerance, to support regulation and reorganization; and (4) support integration by facilitating verbal reflection on emerging bodily experience and linking it with autobiographical memory and meaning, contributing to increased flexibility, affect regulation, agency, and self-integration.

### 7.1. Future Directions

Future directions include empirical investigation of movement-related mechanisms involved in emotional memory encoding, reinstatement, and updating. Experimental studies should examine whether movement configurations present during emotionally salient experiences are reinstated during later retrieval and whether movement variation during reactivation contributes to memory updating. Neuroimaging and psychophysiological research may further clarify the involvement of sensorimotor, interoceptive, salience, autobiographical, and corticocerebellar systems in these processes. Clinical and longitudinal studies are also needed to examine how early movement-anchored emotional organizations relate to later affect regulation, agency, self-experience, interpersonal functioning, and response to movement-based therapeutic interventions. Future experimental studies could operationalize change across complementary levels: movement outcomes (e.g., postural configuration, movement quality, spatial orientation, peripersonal boundaries, action readiness, temporal sequencing, and movement variability), physiological indices (e.g., heart rate variability, electrodermal activity, and interoceptive measures), and memory-related outcomes (e.g., emotional memory accuracy, autobiographical memory specificity, fear generalization, and changes following memory reactivation). Multimodal designs combining quantified movement and behavioral outcomes with neural recording may be particularly informative. Recent work combining exercise intervention with behavioral and hippocampal neural measures provides a methodological example of multilevel assessment [120]. Across psychological and clinical research, mobile neuroimaging approaches may further support concurrent assessment of movement and neural dynamics during naturalistic embodied behavior, increasing ecological validity [121].

Specific testable hypotheses generated by this framework may include the following complementary empirical routes for testing the framework across experimental, developmental, and clinical levels: (1) movement configurations present during emotional encoding will show partial reinstatement during later retrieval, reflected in sensorimotor neural activity and stronger memory effects under movement-congruent than movement-incongruent retrieval conditions; (2) emotional memory retrieval will be accompanied by systematic changes in movement quality and configuration that are congruent with the remembered experience; (3) longitudinal studies should examine whether early caregiver-infant sensorimotor and affective synchrony will predict later flexibility in bodily, affective, and action-related responses during emotionally salient remembering; and (4) therapeutic change in trauma populations will be associated with reduced bodily re-experiencing symptoms and measurable increases in the flexibility and variability of movement-related responses during emotional memory reactivation, including movement quality, spatial engagement, action readiness, and perceived action possibilities.

### 7.2. Limitations and Ethical Considerations

Limitations include the limited direct empirical evidence currently linking specific movement configurations to emotional memory encoding, reinstatement, and reconsolidation. The construct of movement-anchored emotional memory therefore remains an integrative conceptual framework requiring further operationalization and empirical validation. An additional challenge concerns disentangling movement-specific effects from relational, contextual, attentional, and broader experiential factors involved in therapeutic change. Cultural, developmental, and individual variations in movement expression should also be considered when examining their relation to emotional memory organization.

Alongside these limitations, the framework raises several ethical considerations that merit attention in clinical application. Movement-based interventions may carry risks of retraumatization, particularly when emotionally charged movement patterns are reactivated without sufficient pacing, attunement, and relational regulation. Cultural variation in movement expression, the meaning attributed to touch, and norms around bodily engagement should inform how movement-based approaches are implemented across different populations. Adequate training in somatic and embodied approaches, within a broader clinical framework informed by trauma-sensitive practice, is therefore a prerequisite for responsible implementation.

### 7.3. Conclusions

In conclusion, movement-anchored emotional memory offers an integrative perspective for understanding how emotionally significant experiences may be organized through bodily action and carried forward across time. By specifying the temporal, spatial, and action-related contribution of movement within broader embodied memory processes, the framework provides a basis for examining how movement may participate in the encoding, reinstatement, and therapeutic updating of emotional experience. This perspective may contribute to a more embodied understanding of emotional memory and its relevance to psychotherapy, trauma treatment, assessment, and movement-based clinical practice.

## Data Availability

No data was used for the research described in the article.

## Abbreviations

The following abbreviations are used in this manuscript:

PTSD	Post-traumatic stress disorder
DMN	default mode network
ACC	Anterior cingulate cortex
SN	Salience network
AI	Anterior insula
mPFC	Medial prefrontal cortex
DMT	Dance/Movement Therapy
